# Cortical Bone Thickness and Success of Temporary Anchorage Devices in the Mandibular Buccal Shelf: A Retrospective Cone Beam Computed Tomography Study

**DOI:** 10.7759/cureus.115283

**Published:** 2026-08-27

**Authors:** Sushil Bhagwan Mahajan, Vinay V Bedre, Trilok Shrivastav, Barun Kumar, Amarthaluri Avinash, Sreelatha Sadasivan

**Affiliations:** 1 Department of Orthodontics and Dentofacial Orthopedics, People’s College of Dental Sciences and Research Centre, Bhopal, IND; 2 Department of Oral and Maxillofacial Surgery, Bharati Vidyapeeth (Deemed to be University) Dental College and Hospital, Navi Mumbai, IND; 3 Department of Prosthodontics, St. Joseph's Dental College, Eluru, IND; 4 Department of Oral and Maxillofacial Surgery, Sri Sai College of Dental Surgery, Vikarabad, IND

**Keywords:** cone beam computed tomography, cortical bone thickness, mandibular buccal shelf, orthodontic anchorage, temporary anchorage devices

## Abstract

Introduction: Temporary anchorage devices (TADs) have revolutionized orthodontic treatment by providing reliable skeletal anchorage; however, their success on the mandibular buccal shelf remains highly dependent on local bone architecture. This study aimed to evaluate the success rate of TADs placed on the mandibular buccal shelf and to determine optimal cortical and total bone thickness thresholds using preoperative cone beam computed tomography to achieve high success rates.

Materials and methods: This retrospective cohort study included 90 patients aged 18-35 years with an average growth pattern with 180 TADs placed bilaterally in the mandibular buccal shelf area. Cone beam computed tomography scans were analyzed for cortical bone thickness, total bone thickness, and cortical-to-cancellous ratio in the first and second molar regions at depths of 4, 8, and 12 mm from the cemento-enamel junction. Two independently calibrated examiners performed the measurements. Statistical analyses included receiver operating characteristic curves, binary logistic regression, and Kaplan-Meier survival analysis.

Results: The overall success rate of TAD was 82.2% (148/180). The second molar region at 8 mm depth showed the highest cortical bone thickness (5.1 ± 1.3 mm) and yielded the best predictive performance (area under curve = 0.82). A cortical bone thickness threshold of 4.9 mm at this site resulted in a 93.6% success rate. Success was significantly higher with larger device diameter (2.1 ± 0.3 mm vs 1.8 ± 0.4 mm) and higher insertion torque (24.1 ± 5.2 Ncm vs 15.8 ± 4.5 Ncm). Kaplan-Meier analysis revealed 96.9% survival at 24 weeks when the cortical thickness was ≥4.9 mm compared to 72.0% when the cortical thickness was below this threshold (log-rank p < 0.001). Multivariate regression confirmed cortical thickness, total bone thickness, insertion site, device dimensions, and torque as independent predictors.

Conclusion: Preoperative assessment of cortical bone thickness using cone beam computed tomography provides valuable guidance for selecting optimal insertion sites for TADS in the mandibular buccal shelf. Incorporating this approach into routine treatment planning may improve the predictability and clinical success of orthodontic anchorage.

## Introduction

Temporary anchorage devices (TADs) have become essential tools in contemporary orthodontics, providing reliable skeletal anchorage for tooth movement without patient compliance. These miniature implants are particularly valuable in complex cases that require absolute anchorage, such as intrusion, distalization, and correction of vertical discrepancies [1]. Among various insertion sites, the mandibular buccal shelf has gained popularity owing to its accessible location and sufficient bone volume [2]. However, despite technological advancements, TAD failure remains a clinical concern, with reported failure rates ranging from 7.3% for miniplates to 10.5% for palatal implants [3]. Failure of treatment can prolong treatment, increase costs, and necessitate additional procedures.

The primary stability and long-term success of TADs depend on multiple factors, including patient-related variables (age, sex, skeletal pattern), device characteristics (length and diameter), insertion technique, and local bone quality [4]. Cortical bone thickness and total bone thickness are considered critical determinants of mechanical retention and resistance to orthodontic forces. A thinner cortical bone has been associated with a higher failure risk due to inadequate primary stability and increased susceptibility to micromotion [5]. Cone beam computed tomography (CBCT) offers three-dimensional visualization and accurate measurement of bone dimensions at potential insertion sites, enabling preoperative assessment that conventional two-dimensional radiographs cannot provide [6].

Previous studies have investigated various risk factors for TAD failure, yet few have established site-specific quantitative thresholds for bone thickness that reliably predict high success rates on the mandibular buccal shelf [5]. Most research has focused on general success rates or qualitative assessments rather than precise cut-off values derived through robust statistical methods [3-5]. Furthermore, limited data exists on the combined predictive value of cortical and total bone thickness across different insertion depths and molar regions using survival analysis.

The aim of this retrospective study was to evaluate the success rate of TADs placed on the mandibular buccal shelf and to determine the optimal cortical and total bone thickness thresholds for achieving high success rates using preoperative CBCT. The specific objectives were (1) to identify independent predictors of TAD failure by multivariable logistic regression, (2) to derive site- and depth-specific cortical and total bone thickness thresholds on preoperative CBCT that are associated with an observed success rate of at least 90%, using receiver operating characteristic curve analysis, and (3) to compare time-to-failure according to cortical bone thickness using Kaplan-Meier survival analysis.

## Materials and methods

Study design

This retrospective cohort study was conducted at the Department of Orthodontics and Dentofacial Orthopedics, People’s College of Dental Sciences and Research Centre, Bhopal, Madhya Pradesh, India. Data were collected and analyzed in June 2023 from the archived records of patients treated between June 2020 and May 2023. Ethical approval was obtained from the Institutional Ethics Committee (PCDS/IEC/2023/5/106) of the college. Patient confidentiality was maintained by anonymizing the data.

Eligibility criteria

Patient records were included in the study after screening the archival data. The inclusion and exclusion criteria are summarized in Table 1.

**Table 1:** Inclusion and exclusion criteria for study participants. TADs = temporary anchorage devices, CBCT = cone beam computed tomography

Inclusion criteria	Exclusion criteria
Aged 18–35 years	Craniofacial syndromes
Presented with an average growth pattern as determined by mandibular plane angle (SN-GoGn of 28-36^0^)	Congenital anomalies
Had good oral hygiene	History of surgery, trauma, or pathology in the mandibular region
Received orthodontic treatment with TADs in the mandibular buccal shelf	Poor‑quality CBCT scans
Had high‑quality pre‑treatment CBCT scans within three months before insertion	Incomplete clinical records
Had complete clinical records	Pregnancy or systemic conditions affecting bone metabolism

Sample size estimation

Sample size estimation was performed using the G*Power software (version 3.1.9.7; Heinrich Heine University, Düsseldorf, Germany). To identify predictors of TAD failure using binary logistic regression, sample size was calculated based on the "events per variable" (EPV) rule. With an anticipated TAD success rate of approximately 75-80% (based on reported mandibular TAD success rates of ~79-87%) and inclusion of 10 predictor variables, a minimum of eight to 10 events per predictor variable was recommended [7]. The estimated sample size was 125. To account for 10% of the incomplete records and to increase the power of the study, the final target sample was 180 TADs.

CBCT imaging protocol

All CBCT scans were acquired using a CBCT unit (NewTom VGi evo, NewTom, Cefla S.C., Imola, Italy) with standardized imaging parameters: a field of view (FOV) of 16 × 12 cm or larger, an isotropic voxel size of 0.3 mm, tube voltage of 110 kVp, tube current of 4-6 mA, and an exposure time of 3.6-5.4 s. During image acquisition, the patients were positioned with the Frankfort horizontal plane parallel to the floor and instructed to maintain maximum intercuspation (centric occlusion).

CBCT measurements and predictor variables

**Figure 1:**
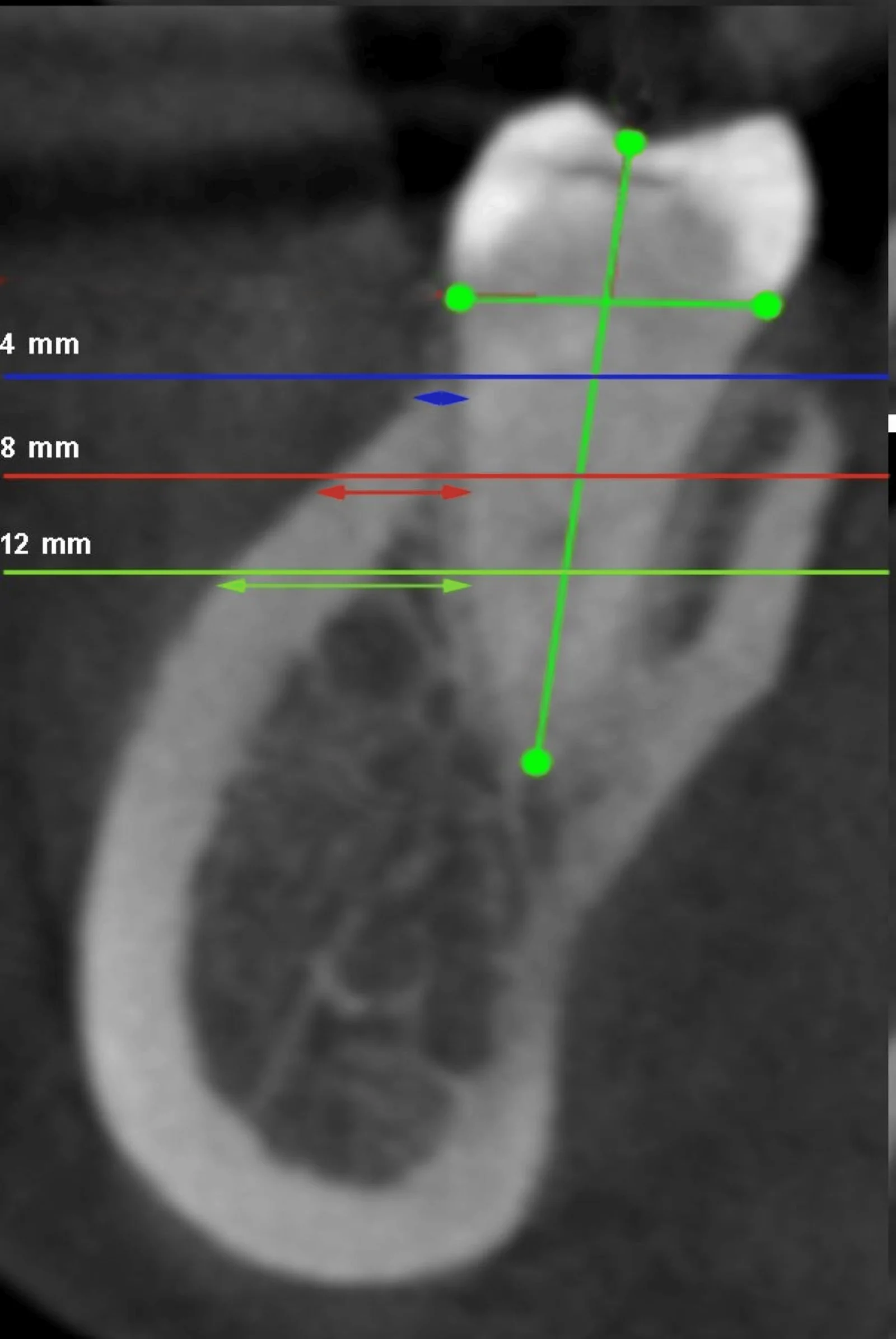
Cone beam computed tomography (CBCT) image of mandibular first molar showing bone thickness of buccal shelf area at 4, 8 and 12 mm distance from the cemento-enamel junction. Blue horizontal plane: 4 mm distance from cemento-enamel junction, Blue arrow: bone thickness of buccal shelf area. Red horizontal plane: 8 mm distance from cemento-enamel junction, Red arrow: bone thickness of buccal shelf area. Green horizontal plane: 12 mm distance from cemento-enamel junction, Green arrow: bone thickness of buccal shelf area. Original CBCT image of the patient from the study sample.

Predictor variables included cortical bone thickness, total bone thickness, and cortical-to-cancellous ratio measured at two insertion sites (mesiobuccal root of the first molar and distobuccal root of the second molar) and at three depths (4 mm, 8 mm, and 12 mm apical to the cemento-enamel junction). The cortical bone thickness was measured as the perpendicular distance from the outer surface of the buccal cortical plate to the cancellous bone interface. The total bone thickness was measured as the linear distance from the outer surface of the buccal cortical plate to the outer surface of the lingual cortical plate, taken perpendicular to the vertical axis of the mandibular body at each specified depth (Figure 1). The cortical-to-cancellous ratio was calculated as the cortical bone thickness divided by (total bone thickness minus the cortical bone thickness). Additional clinical predictor variables were age, sex, skeletal pattern, TAD length and diameter, and insertion torque. Measurements were performed using OnDemand3D software (Cybermed Inc., Daejeon, Korea) and InVivo 6 software (Anatomage Inc., Santa Clara, CA, USA).

Calibration and reliability testing

Two independent examiners (BK and AA) from different institutions performed all radiographic measurements while blinded to clinical outcomes. Both examiners underwent rigorous calibration training using 25 randomly selected scans. Inter-observer and intra-observer reliabilities were assessed using intraclass correlation coefficients (ICCs), yielding excellent results (inter-observer ICC 0.91-0.96; intra-observer ICC 0.94-0.98).

Outcome variables

The primary outcome was TAD failure, defined as mobility, loosening, or loss of the device at any time during treatment, including early failure (within four weeks) and late failure (after four weeks). Success was defined as stable retention of the device throughout the intended orthodontic treatment period, without clinical mobility or radiographic signs of pathology. Secondary outcomes included insertion torque (in Ncm) and survival time (in weeks) from placement to treatment failure or completion.

Statistical analysis

All statistical analyses were performed on coded data using IBM SPSS Statistics for Windows (version 25.0; IBM Corp., Armonk, NY, USA). Continuous variables are summarized as mean ± standard deviation (SD), while categorical variables are expressed as frequencies and percentages. Baseline comparisons between the TAD's success and failure groups were conducted using independent-samples t-tests (or Mann-Whitney U test for non-normal data) for continuous variables and Chi-square or Fisher's exact tests for categorical variables. Receiver operating characteristic (ROC) curve analysis with Youden's index identified optimal cortical and total bone thickness thresholds for achieving ≥90% success with TADs. Binary logistic regression (forced entry method) was performed to identify independent predictors of failure, and model fit was assessed using the Hosmer-Lemeshow test and Nagelkerke R². Time-to-failure was analyzed using Kaplan-Meier survival curves and the log-rank test. For the multivariable logistic regression, only the cortical and total bone thickness values corresponding to the actual insertion site and planned insertion depth of each TAD were used. Youden’s index identified the optimal ROC cut-off; the observed success rate among devices above that cut-off was then calculated, and thresholds achieving ≥90% success were reported. One hundred and eighty TADs were placed in 90 patients; patient-level variables were analysed on the unique 90 individuals and device-level variables on the full set of 180 TADs. Statistical significance was set at two-sided p < 0.05.

## Results

A total of 180 TADs were evaluated in 90 patients where TADs were placed bilaterally in the mandibular buccal shelf area, of which 148 (82.2%) were successful and 32 (17.8%) were unsuccessful. Patients in the success group were significantly older than those in the failure group (29.1 ± 9.8 vs. 25.3 ± 8.2 years; p = 0.043). Sex distribution and skeletal classification did not differ significantly between groups (p = 0.956 and p = 0.053, respectively). The success of TADs was significantly associated with the insertion site, with a greater proportion of successful devices being placed in the second molar region than in the first molar region (p = 0.004). Successful TADs also demonstrated significantly greater implant length (7.9 ± 1.1 vs. 7.3 ± 1.4 mm; p = 0.010), implant diameter (2.1 ± 0.3 vs. 1.8 ± 0.4 mm; p < 0.001), insertion torque (24.1 ± 5.2 Ncm vs. 15.8 ± 4.5 Ncm; p < 0.001), and follow-up duration (27.1 ± 7.9 vs. 23.2 ± 8.8 weeks; p = 0.018) (Table 2). The shorter follow-up duration in the failure group is explained by the fact that observation of each device was terminated at the time of failure; successful devices continued to be followed until completion of orthodontic treatment. This difference therefore reflects early event occurrence rather than survivorship bias or differential loss to follow-up.

**Table 2:** Descriptive characteristics of the study population according to temporary anchorage device (TAD) outcome. Data are presented as mean ± standard deviation or number (percentage), independent-samples t-test was used for continuous variables and Chi-square test for categorical variables, effect sizes are presented as Cohen's d, Phi coefficient, or Cramer's V where appropriate, *p < 0.05 was considered statistically significant, SD = standard deviation, Ncm = Newton centimeter, df = degree of freedom. Age, sex and skeletal class were analysed at the patient level (n = 90). All other variables were analysed at the device level (N = 180 TADs).

Characteristic		Overall (N = 180 TADs / 90 patients)	TAD success (n = 148)	TAD failure (n = 32)	Test value (df)	p-value	Effect size
Age, Mean ± SD	Years	28.4 ± 5.2	29.1 ± 5.0	25.3 ± 4.8	t (88) = 3.420	0.001*	d = 0.760
Sex, n (%)	Female	51 (56.7%)	42 (56.8%)	9 (56.3%)	χ² (1) = 0.003	0.956	φ = 0.004
Skeletal, n (%)	Class I	29 (32.2%)	26 (35.1%)	3 (18.8%)	χ² (2) = 5.874	0.053	V = 0.181
	Class II	36 (40.0%)	29 (39.2%)	7 (43.8%)			
	Class III	25 (27.8%)	19 (25.7%)	6 (37.5%)			
Insertion site, n (%)	First molar region	85 (47.2%)	64 (43.2%)	21 (65.6%)	χ² (1) = 8.081	0.004*	φ = 0.212
	Second molar region	95 (52.8%)	84 (56.8%)	11 (34.4%)			
TAD length, Mean ± SD	mm	7.8 ± 1.2	7.9 ± 1.1	7.3 ± 1.4	t (178) = 2.596	0.010*	d = 0.524
TAD diameter, Mean ± SD	mm	2.0 ± 0.4	2.1 ± 0.3	1.8 ± 0.4	t (178) = 4.330	<0.001*	d = 0.874
Insertion torque, Mean ± SD	Ncm	22.6 ± 5.8	24.1 ± 5.2	15.8 ± 4.5	t (178) = 8.491	<0.001*	d = 1.714
Follow-up duration, Mean ± SD	Weeks	26.4 ± 8.2	27.1 ± 7.9	23.2 ± 8.8	t (178) = 2.378	0.018*	d = 0.480

Cortical bone thickness, total bone thickness, and the cortical-to-cancellous ratio progressively increased from 4 mm to 12 mm apical to the cemento-enamel junction at both insertion sites. At the first molar region, cortical bone thickness increased from 2.8 ± 0.9 mm to 4.8 ± 1.4 mm, whereas total bone thickness increased from 7.2 ± 1.8 mm to 9.4 ± 2.3 mm. Similarly, at the second molar region, cortical bone thickness increased from 3.2 ± 1.0 mm to 5.4 ± 1.5 mm, while total bone thickness increased from 7.8 ± 1.9 mm to 10.6 ± 2.4 mm. The cortical-to-cancellous ratio also increased with increasing depth, reaching 1.04 at the 12-mm level in both regions (Table 3).

**Table 3:** Cone beam computed tomography (CBCT)-derived bone parameters according to insertion site and measurement depth. Data are presented as mean ± standard deviation, measurements were obtained at 4, 8, and 12 mm apical to the cemento-enamel junction in the first and second molar regions, CEJ = cemento-enamel junction, SD = standard deviation.

Site	Depth (mm from CEJ)	Cortical thickness (mm) Mean ± SD	Total thickness (mm) Mean ± SD	Cortical-to-cancellous ratio Mean ± SD
First molar region	4	2.8 ± 0.9	7.2 ± 1.8	0.64 ± 0.28
	8	4.2 ± 1.2	8.9 ± 2.1	0.89 ± 0.35
	12	4.8 ± 1.4	9.4 ± 2.3	1.04 ± 0.42
Second molar region	4	3.2 ± 1.0	7.8 ± 1.9	0.70 ± 0.30
	8	5.1 ± 1.3	10.2 ± 2.2	1.00 ± 0.38
	12	5.4 ± 1.5	10.6 ± 2.4	1.04 ± 0.40

The site-specific threshold values associated with the high success of the TADs are presented in Table 4. At the first molar region, cortical bone thickness thresholds of 4.3 mm and 4.8 mm at the 8-mm and 12-mm levels corresponded to success rates of 91.6% and 90.3%, respectively. At the second molar region, cortical bone thickness thresholds of 4.9 mm and 5.3 mm achieved success rates of 93.6% and 91.5%, respectively. Corresponding total bone thickness thresholds were 9.2 mm and 9.8 mm in the first molar region and 10.4 mm and 10.8 mm in the second molar region. Threshold values at the 4-mm level did not achieve a success rate of 90% at either insertion site (Table 4).

**Table 4:** Site-specific threshold values associated with high success of temporary anchorage devices. Threshold values were determined using receiver operating characteristic curve analysis and Youden index, data are presented as threshold values, achieved success rates, and 95% confidence intervals (CI), CEJ = cemento-enamel junction.

Insertion site	Depth (mm from CEJ)	Minimum cortical thickness (mm)	Minimum total thickness (mm)	Minimum cortical-to-cancellous ratio	Achieved success rate (%)	95% CI
First molar region	4	≥ 3.5 mm	≥ 8.0 mm	≥ 0.75	85.0	73.9-91.9
	8	≥ 4.3 mm	≥ 9.2 mm	≥ 0.90	91.6	83.0-96.1
	12	≥ 4.8 mm	≥ 9.8 mm	≥ 0.95	90.3	80.8-95.1
Second molar region	4	≥ 3.8 mm	≥ 8.5 mm	≥ 0.80	87.5	77.5-93.6
	8	≥ 4.9 mm	≥ 10.4 mm	≥ 1.00	93.6	85.9-97.2
	12	≥ 5.3 mm	≥ 10.8 mm	≥ 1.00	91.5	83.6-96.3

**Figure 2:**
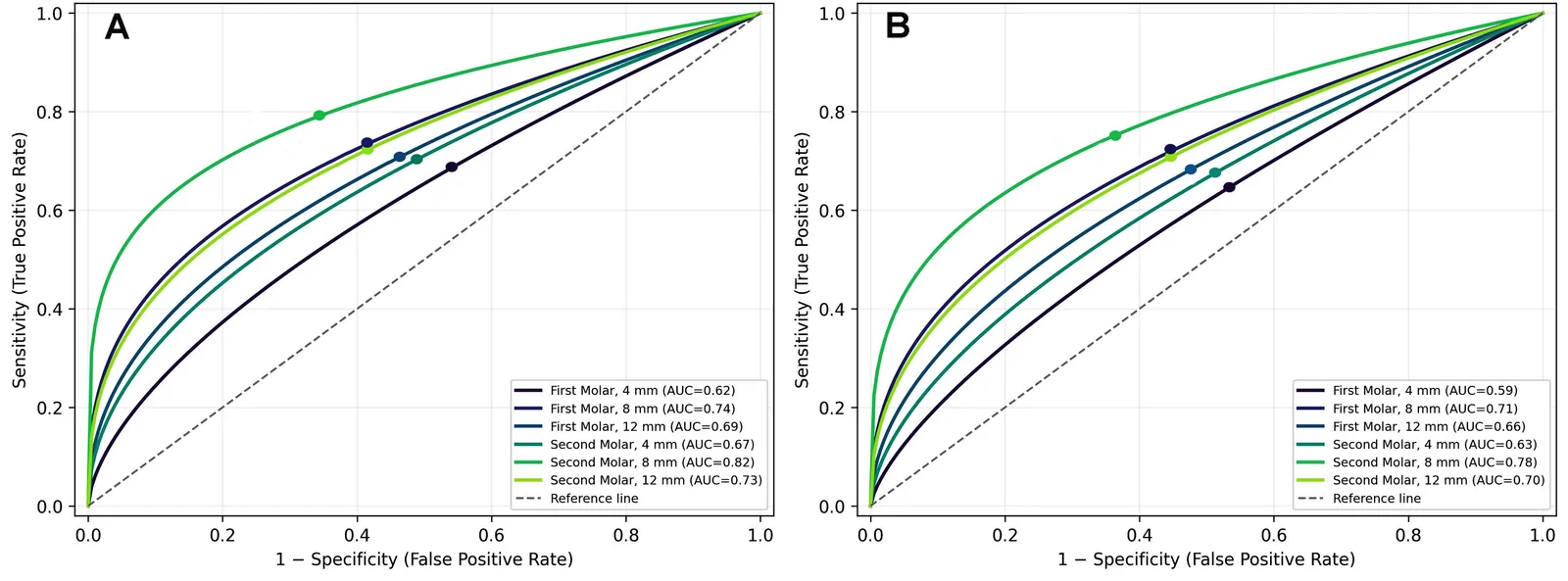
Receiver operating characteristic curves for (A) cortical bone thickness and (B) total bone thickness in predicting the success of temporary anchorage devices. Receiver operating characteristic curves showing the diagnostic performance of cortical bone thickness and total bone thickness measured at different insertion sites and depths for predicting the success of temporary anchorage devices, AUC = area under the curve.

Cortical bone thickness measured at the second molar region, 8 mm apical to the cemento-enamel junction, demonstrated the highest diagnostic performance for predicting the success of TADs (area under the curve = 0.82), followed by cortical bone thickness in the first molar region at the same depth (area under the curve = 0.74). Total bone thickness showed a similar trend, with the highest predictive accuracy observed in the second molar region, 8 mm from the cemento-enamel junction (area under the curve = 0.78). The optimal cut-off values identified by ROC curve analysis were 4.9 mm for cortical bone thickness and 10.4 mm for total bone thickness, with corresponding sensitivities of 79.1% and 75.0% and specificities of 75.0% and 71.9%, respectively (Figure 2).

Multivariate binary logistic regression analysis demonstrated that cortical bone thickness, total bone thickness, implant diameter, implant length, insertion torque, and insertion site were independent predictors of TAD failure (all p < 0.05), whereas age and sex were not independently associated with failure (Table 5).

**Table 5:** Multivariable binary logistic regression analysis of independent predictors of temporary anchorage device (TAD) failure. Binary logistic regression analysis was performed using the forced-entry method, *p < 0.05 was considered statistically significant, SE = standard error, CI = confidence interval, OR = odds ratio.

Predictor variable	β coefficient	SE	Wald χ²	df	p-value	Adjusted Odds Ratio (95% CI)
Cortical thickness (mm)	-0.162	0.042	14.88	1	<0.001*	0.850 (0.784-0.922)
Total thickness (mm)	-0.098	0.035	7.84	1	0.005*	0.907 (0.847-0.971)
Age (years)	-0.019	0.015	1.60	1	0.206	0.981 (0.953-1.010)
Sex (Male vs Female)	0.087	0.245	0.13	1	0.721	1.091 (0.676-1.762)
TAD diameter (mm)	-0.512	0.148	11.97	1	0.001*	0.599 (0.448-0.801)
TAD length (mm)	-0.124	0.058	4.57	1	0.033*	0.883 (0.788-0.989)
Insertion torque (Ncm)	-0.328	0.089	13.58	1	<0.001*	0.720 (0.605-0.858)
Insertion site (Second vs First molar)	-0.482	0.218	4.89	1	0.027*	0.617 (0.402-0.946)
Constant	8.234	1.892	18.94	1	<0.001*	-

**Figure 3:**
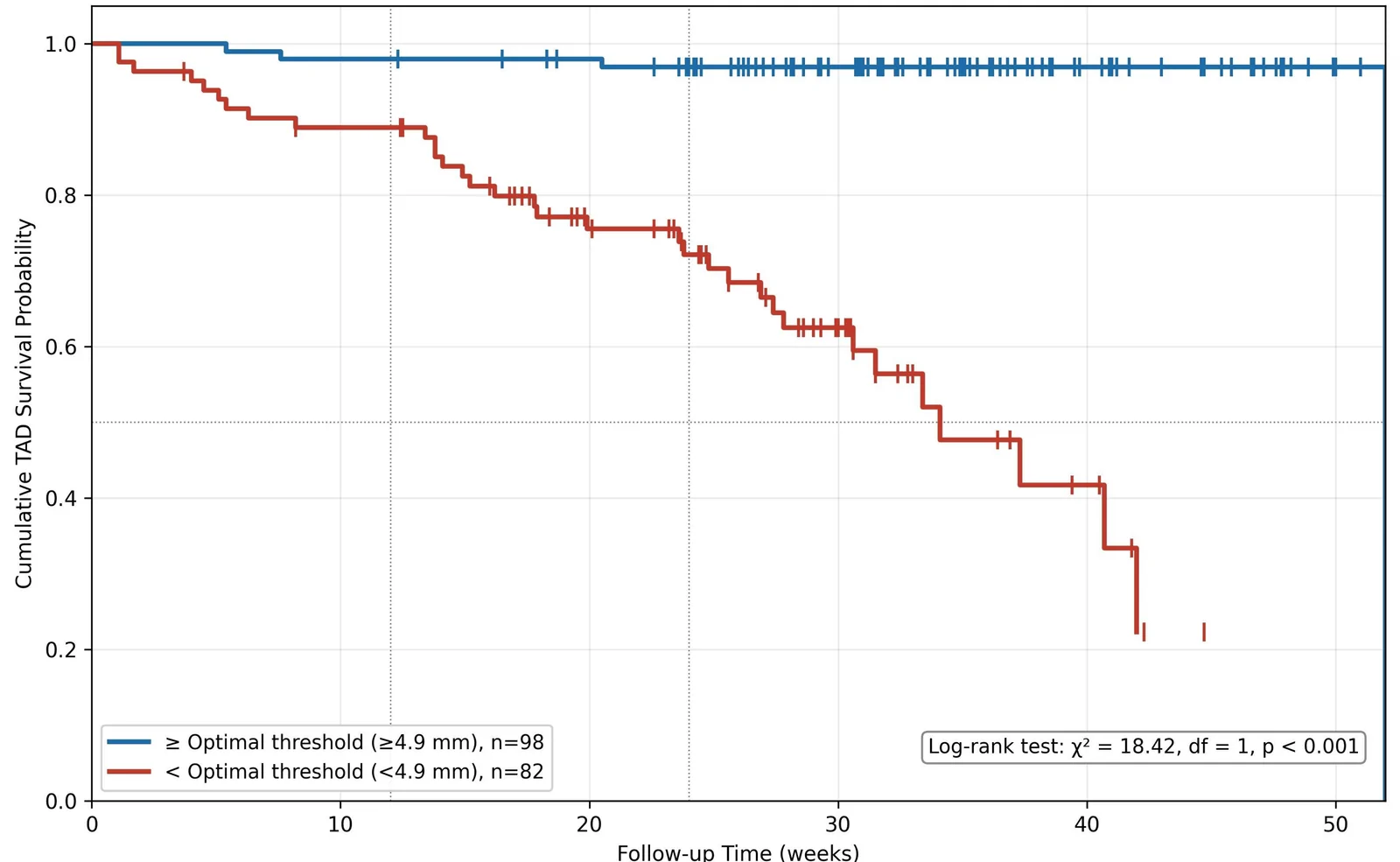
Kaplan-Meier survival curves of temporary anchorage devices according to cortical bone thickness. Kaplan-Meier curves comparing the cumulative survival of temporary anchorage devices placed in cortical bone thickness ≥4.9 mm and <4.9 mm, p value was calculated using the log-rank test, TAD = temporary anchorage device.

Kaplan-Meier survival analysis demonstrated significantly greater cumulative survival of TADs placed in the cortical bone measuring ≥4.9 mm than those placed in the cortical bone measuring <4.9 mm (log-rank χ² = 18.42, p < 0.001). In the group with a cortical bone thickness ≥4.9 mm (n = 98), the median survival time was not reached during follow-up, with cumulative survival probabilities of 98.0% at 12 weeks and 96.9% at 24 weeks. In contrast, the group with cortical bone thickness <4.9 mm (n = 82) had a median survival time of 38.2 weeks (95% confidence interval: 34.6-41.8 weeks), with cumulative survival probabilities of 87.8% at 12 weeks and 72.0% at 24 weeks (Figure 3).

## Discussion

The identification of cortical bone thickness as a dominant predictor of TAD success on the mandibular buccal shelf provides important mechanistic insight into the determinants of skeletal anchorage stability. The superior performance of the second molar region at 8 mm depth likely arises from its unique anatomical configuration, characterized by a denser cortical architecture and optimal load-bearing capacity away from the root apices. Nookala et al. [8] demonstrated that the distal aspect of the mandibular second molar, particularly 8-12 mm apical to the cemento-enamel junction, provides the greatest cortical and total bone thickness for buccal shelf mini-screw placement. Nucera et al. [9] reported that the mandibular buccal shelf adjacent to the second molar provided adequate bone volume and cortical bone thickness for extra-alveolar mini-screw insertion.

This finding extends beyond simple dimensional associations by demonstrating how site-specific bone morphology influences primary mechanical retention and long-term biological integration. In high-density cortical bones, improved thread engagement reduces interfacial stress concentrations and micromotion, thereby limiting the activation of inflammatory pathways that compromise secondary stability [10]. Such observations align with finite element studies showing that cortical thickness modulates stress distribution more profoundly than does total bone volume alone, offering a plausible explanation for the differential outcomes observed between insertion sites and depths [11,12].

These results advance the literature in several ways. Although previous investigations have consistently reported higher failure rates in the mandible than in the maxillary sites, few have moved beyond broad associations to establish clinically actionable, depth-specific thresholds using rigorous ROC curve methodology [13,14]. The present work complements studies such as those by Aly et al. [7], who highlighted insertion torque and bone quality, yet provided more granular, preoperative planning parameters that can be readily translated into practice.

Motoyoshi et al. [15] demonstrated that mini-implants placed in cortical bone ≥1.0 mm thick had significantly higher success rates than those inserted in thinner cortical bone, emphasizing cortical bone thickness as a critical determinant of implant stability. They further reported that excessive insertion torque should be avoided despite thicker cortical bone, and recommended an insertion torque of approximately 8-10 Ncm to optimize clinical success. It should be noted that the cortical-thickness threshold and insertion-torque values reported by Motoyoshi et al. [15] were derived from inter-radicular mini-implants placed in relatively thin alveolar bone. The mandibular buccal shelf, by contrast, presents substantially thicker cortical bone; consequently a higher absolute cortical-thickness threshold (4.9 mm) and higher insertion torques are observed. While an upper torque limit that could produce compression necrosis may still exist, the association between torque and success appeared monotonic within the range recorded in the present buccal-shelf cohort.

Similarly, Leo et al. [16] concluded that adequate cortical and total bone thicknesses are fundamental prerequisites for achieving stable skeletal anchorage, while acknowledging that factors such as insertion torque, implant design, and surgical technique also influence clinical outcomes. Kaplan-Meier analysis further enriches this understanding by illustrating that the protective effect of adequate cortical thickness persists over time, suggesting that early stability translates into sustained resistance against fatigue failure under prolonged orthodontic loading. This temporal dimension is particularly relevant in contemporary orthodontics, where treatment durations are often extended in complex cases that require absolute anchorage. Umeh et al. [17] reported a six-month survival rate of 88.9% for TADs, with the majority of failures occurring within the first month after placement. The authors found no significant association between the survival of TADs and patient age, sex, implant site, side, dental arch, or implant length, suggesting that early stability is more dependent on local biomechanical and biological conditions than on demographic or implant-related variables.

Clinically, these findings have significant implications in treatment planning and patient selection. Routine preoperative CBCT evaluation with attention to cortical thickness at the second molar 8 mm site can facilitate risk stratification, allowing clinicians to optimize insertion parameters, select appropriate device dimensions, or consider alternative anchorage strategies when bone conditions are suboptimal. Such an approach has the potential to reduce failure rates, minimize unanticipated treatment interruptions, and enhance overall treatment efficiency. Moreover, in the era of personalized orthodontics, these thresholds support shared decision-making by providing objective data to counsel patients regarding expected success probabilities. The independent contributions of device diameter and insertion torque further suggest that a multifaceted strategy of combining optimal site selection with appropriate hardware and techniques offers the best chance for predictable outcomes.

This study has several limitations. Its retrospective design carries the inherent risks of selection and information bias. The cortical-thickness thresholds were derived and evaluated in the same cohort without internal validation (bootstrap or hold-out), so the reported success rates may be optimistic. Multiple ROC analyses were performed across site-depth combinations; no formal multiplicity adjustment was applied. The number of failure events relative to the number of predictors in the multivariable model is modest, which can affect coefficient stability. Insertion torque, although clinically relevant, lies on the causal pathway between cortical thickness and success and was not available preoperatively. The lack of quantitative bone-density assessment and the restriction to patients with average growth patterns limit generalisability. Unmeasured variables such as oral hygiene, parafunctional habits and soft-tissue thickness may also have influenced outcomes.

Future research should prioritize prospective multicenter designs incorporating advanced imaging analytics, artificial intelligence-assisted measurements, and long-term follow-up. Exploration of combined radiographic and clinical prediction models, as well as the impact of the insertion angle and surface modifications, would further strengthen the evidence base. In conclusion, by establishing robust, site-specific cortical bone thickness thresholds, this study meaningfully contributes to the optimization of TAD-assisted orthodontics and underscores the transformative potential of three-dimensional imaging in enhancing anchorage predictability.

## Conclusions

This study demonstrates a consistent association between greater cortical bone thickness and higher success of TADs placed in the mandibular buccal shelf. Among the sites and depths examined, the second-molar region provided the most favourable bone dimensions and the strongest relationship with device survival. The site- and depth-specific findings are exploratory, were derived from a single cohort, and require external validation before clinical adoption. Preoperative CBCT assessment of cortical thickness may assist treatment planning when buccal-shelf anchorage is considered, but the present observational design cannot confirm that such imaging improves outcomes relative to conventional planning. Prospective studies in more diverse populations are warranted.
